# A New Model of Multiple Intelligence for Teaching English Informatics in the IoT Scenario

**DOI:** 10.1155/2022/5642284

**Published:** 2022-06-21

**Authors:** Yang Ning

**Affiliations:** School of Education, Xi'an Fanyi University, Xi'an, Shaanxi 710105, China

## Abstract

This paper presents an in-depth study on the new mode of intelligent multidistance teaching of English with the help of virtual scenes of the Internet of Things. The virtual simulation technology is integrated into the traditional IoT teaching, and the professional education of IoT application technology is tapped; from the analysis of the current situation of IoT skills teaching and the feasibility of carrying out virtual simulation teaching, the “four-driven” design principle is proposed, and the teaching design is combined with the virtual simulation technology teaching, and the case design of the skill-based virtual simulation technology teaching of experience, demonstration, interaction, and assessment in the virtual environment is given. This paper presents the case design of virtual simulation teaching in a virtual environment with experience, demonstration, interaction, and assessment, and the multidimensional effect evaluation of IoT skills teaching and researches the application of virtual simulation to IoT skills teaching through the above four aspects. In this paper, a framework for distributed collaborative computing is built using an asynchronous message queue MQ, which enables multiple nodes to serve a task through task splitting. The DeepCluster module can effectively cluster the time series by deep representation learning and obtain the typical variation of time series patterns. In the task offloading module of the framework, a task offloading decision algorithm based on a value-constrained multi 0–1 backpacking model is designed to minimize task processing latency with an optimal offloading solution. The system test results show that the proposed distributed computing framework and offloading decision algorithm can significantly reduce the processing latency of large tasks.

## 1. Introduction

With the rapid development of science and technology and the advent of the information age, various new technologies and means have emerged. Knowledge is no longer an unchanging objective reality, but a constantly generated and changing existence, requiring individuals to constantly reconstruct their knowledge systems with an open mind [1]. The development of the times has transformed individuals and influenced how international competition takes place, and the needs of countries for talent are gradually changing, with more emphasis on innovative talent with the ability to investigate and practice. Information technology is the main theme of the twenty-first century. Information technology is changing people's lives in all aspects of work, learning, and everyday communication. The use of information technology in teaching and learning is an inevitable trend in the future development of education, as it can provide rich resources for teaching and learning to make the teaching situation more realistic and the teaching process more efficient. This is an important issue in front of the basic education management authorities and teachers of all subjects. The MRE performance of the Holt–Winter method is poor, up to 12.77%, while the proposed method is 4.42%, which is also consistent with the previous ADF test results.

The age of information technology and the widespread use of Internet technology have already made the advantages of the Internet in the field of education very evident, and as “Internet+” thinking is accepted by more people, the new model of “Internet English education” will be more. This will effectively promote the development of English informatization teaching in colleges and universities and open a richer path of informatization teaching [2]. In the traditional English education environment, teaching activities are relatively closed, with teachers, teaching aids, classrooms, and students being confined to a specific area of the classroom, and formal teaching practice is strictly limited by class time [3]. English teachers must choose a relatively closed teaching format to control classroom discipline and emphasize their teaching authority.

## 2. Related Work

In terms of application, intelligent teaching and learning activities appeared earlier in some higher education institutions in the United States, such as Carnegie Mellon University Saddleback College, California, Pennsylvania State University, Austin Peay State University, and so on. Students in these universities can use smart terminals to carry out learning activities anytime and anywhere [4]. In a smart teaching IoT environment, student behavior and data in the smart campus environment are stored in the IoT cloud, and teachers, course managers, or research scholars, for example, can directly or indirectly access the data from the cloud to develop smart, personalized teaching and learning activities for students [5].

The University of Reading in the UK has studied student interaction in smart classrooms, the DEBBIE project at DePaul University has developed an automated system for classroom note-taking, and Arizona State University's smart classrooms use PDAs, context-sensitive middleware based on ubiquitous computing and web technologies. In the mid to late 2010s, IT-assisted teaching entered a new phase of computer-assisted language teaching; the rapid development of IT-based on computer technology, network technology, and modern communication technology has greatly changed the traditional teaching model [6]. Yang et al. believe that wisdom education is a product of economic globalization, technological change, and knowledge explosion and is also an inevitable stage and a new realm and demand for the development of education informatization, which requires a wisdom learning environment as the technical support, wisdom learning as the fundamental cornerstone, and wisdom pedagogy as the catalytic facilitator [7]. Gao et al. also argue from multiple perspectives about the strategies, approaches, elements, and technologies needed to realize the concept of wisdom education in the process of education informatization [8]. There are important functional differences and special connections between teaching and learning: learning as the ontology (purpose), teaching as a condition (means). In other words, “learning is the center, teaching is for learning.”

Although the expressions for online learning spaces vary, researchers have emphasized that learning spaces supported by information technology tools provide a dedicated online learning environment for teachers and students, offering them resources sharing, course learning, online testing, class feedback, and other relevant content [9]. Therefore, the online learning space referred to in this paper is a virtual space created with the help of an online platform to support educational and teaching activities [10]. This space can be used in the English classroom to change the original English teaching model, making English course learning more flexible, free, and interesting. In terms of flexibility, English teachers can change the way they teach according to different teaching contents and make full use of the online learning space platform to support teaching and learning for those aspects of the teaching task that can be achieved through the online environment [11]. In terms of freedom, English teachers can use the e-learning space to meet their daily teaching needs, taking into account the characteristics of the classes they teach and the learning situation, such as independent learning before classes, independent tests, and group discussions in online micro-lessons and micro-videos, making full use of the online platform to expand their knowledge of English cultural background and setting different learning contents and tests for students at different stages to stimulate their participation and enthusiasm in learning. In terms of interest, the novelty of the online learning space and the diversity of pictures and videos can provide students with a different form of teaching from traditional classroom lectures, mobilize students' interest and initiative while they perceive the teaching content and tasks, enable students to realize self-learning, self-improvement, and self-improvement with the help of the online learning platform, and at the same time stimulate their innovative thinking and creative abilities.

## 3. Construction of a Multiple Intelligence Model for English Informatics Teaching Based on IoT Scenarios

### 3.1. The IoT Scenario Model Design

Unlike Internet data such as pictures, voices, videos, and so on, most of the IoT data studied in this paper are generated by humans in their daily activities, so they are affected by many external factors. External factors are the main factors that affect the changes of IoT time series data. Therefore, the periodicity of external factors also leads to the periodicity of time series to a certain extent. For example, under the action of “week,” many IoT time series data have a periodicity of 7 days. However, under the influence of many other external factors as well as random factors, this periodicity can be destroyed and become quasiperiodic. In addition to this, it may also have longer quasiperiods, such as months and years.

Virtual simulation technology is a relatively new technology that has emerged in recent years and can also be called simulation technology. Virtual includes the construction of virtual situations with the help of advanced science and technology, while simulation includes the construction of realistic three-dimensional effects in three-dimensional space, through experience, demonstration, and the operation to complete some complex skills operation points. Of course, these must be built on the rapid development of technology; the current hot augmented reality (AR) technology is also based on this construction; its principle is different from virtual simulation technology, which is to superimpose virtual objects and real environment to the same picture, but it is expensive, not conducive to the current development, and simulation technology and virtual technology are combined after the product, applying them in practical training teaching activities [12]. The combination of simulation and virtual technology can be used in practical training and teaching activities, so it is very promising to use virtual simulation technology mainly in the current teaching design. It creates a good learning environment for students so that no matter what stage of learning they are in, they can choose effective information technology means to obtain, analyze, and comprehensively compare information, learn corresponding professional knowledge of disciplines, and cultivate corresponding practical application ability. The virtual simulation system is based on virtual simulation technology, using relevant hardware equipment to carry out various activities in the virtual world, so that students are in a relatively realistic environment to carry out practical training; the use of virtual simulation system to complete the practical training teaching has a relatively good effect. The virtual simulation system is used to teach students in a relatively realistic environment.

**Figure 1:**
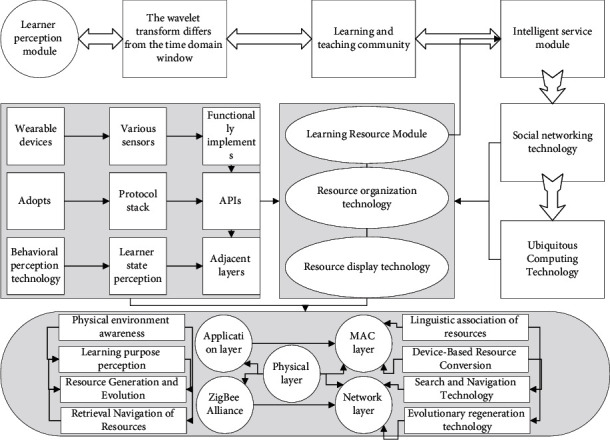
Teaching system and functional model for IoT scenarios.

With the advancement of modern teaching technology, the theory of “blended learning” has gradually replaced constructivism and other theories as one of the important theoretical foundations of educational technology [13]. “The definition of “blended learning” was developed in 2002 by renowned American educators Smith J and Eilert Macieh based on the traditional teaching philosophy and the “E-Learning” (digital learning) learning approach of information and Internet technology. It is based on the concept of “E-Learning” (digital learning) based on traditional teaching concepts and information and Internet technology learning methods. Their quantitative research revolves around humanistic and constructivist theories, among others [14]. Thus, the hybrid theory can be seen as the latest form of constructivist teaching and learning theory. Bruner's structuralist theory of teaching and learning also states that students are active receivers of knowledge and processors of the information they receive. In the decade of the new national curriculum reform, constructivist learning theory once became a common teaching theory used by experts and scholars in academic research and thesis writing. It is in line with the “learning by doing” and “teaching by doing” approach, which mobilizes learners' initiative, active discovery, and interest in learning. The rise of modern distance learning is also complementary to this. Hybrid teaching is a mixture of online and traditional classroom teaching methods to overcome the shortcomings of traditional teaching methods, and the IoT scenario teaching system and functional model are shown in Figure 1. This also means that a single model between learning and teaching is no longer suitable for the development of education and teaching, and diversified learning and teaching methods need to be effectively integrated into daily teaching activities.

As far as the main object of education and teaching is concerned, online learning spaces can provide students with more personalized guidance and intelligent technical support; for example online learning spaces can be used in the process of setting up space resources, which students can access as green resources that reject harmful information on the Internet [15]. This online learning platform, based on the Internet + era and the progress of modern information technology, can improve students' motivation and initiative in learning. As far as teachers, who are the guides of educational and teaching activities, are concerned, the online learning space is not only advantageous in terms of lectures but also has a positive effect in terms of teachers improving their quality and educational and teaching level, in such as online video observation lessons, online teaching, and research. It is also beneficial for teachers to understand and grasp the learning level and academic level of students through the online space so that they can teach according to their ability and make the best use of the situation in their teaching practice.

(1) $$\begin{array}{c} \lambda =\left\{\begin{array}{cc} \frac{T_{\text{RC}}}{T_{\text{RD}}}, & T_{\text{RC}}<T_{\text{RD}},\\ 1, & T_{\text{RC}}\geq T_{\text{RD}}, \end{array}\right.\\ x\left(t:L:l\right)=\left[x\left(x-l\right),\ldots ,x\left(t-L_{\text{l}}\right)\right]. \end{array}$$

It can not only realize the video teaching arranged and arranged by the teacher, but also choose the relevant courses in the space according to personal interests. This online learning platform based on the Internet + era and the progress of modern information technology can improve students' learning enthusiasm and initiative. As mentioned earlier, IoT time series are subject to a wide range of external influences, are more complex, and mostly contain distinct trend, seasonal or cyclical terms, so they are usually nonstationary time series. Although in some IoT scenarios, certain time series may be smooth in the short term; they generally behave nonstationary. In the case of an energy load series in the industrial IoT, for example, the temperature is one of the most important factors affecting energy load, and temperature data generally does not change much in the short term. Therefore, in general, provincial/municipal energy loads remain relatively stable over short periods, such as a day, a week, or even a month, but vary considerably from quarter to quarter.

(2) $$\begin{array}{c} x\left(\left[t^{\text{miss}}\right]\right)=f{\left(x\left(\left[t^{\text{obs}}\right]\right)\right)}^{2}. \end{array}$$

How to utilize the typical features of IoT time-series data and thus provide a panoramic view and a comprehensive analysis method for the development and operation of IoT systems is the focus of this thesis. Time series prediction is a prerequisite for intelligent decision-making, while time-series complementation is the basis for time series analysis based on big data technology. Therefore, this thesis will focus on the research of time series prediction and missing value algorithms based on machine learning to provide a more complete idea for intelligent decision-making of IoT systems. Among them, time series prediction mainly relies on historical time-series data as well as external influence factors to infer the future development trend of data. It is a prerequisite for intelligent decision-making and management and can help people to make reasonable plans to cope with various uncertainties in the future. Time series missing values rely primarily on known measurement data to recover missing data [16]. It is the foundation of big data analytics technology, which ensures the temporal and spatial integrity of time-series data to support various subsequent analytical tasks, such as prediction and classification.

(3) $$\begin{array}{c} F=\arg\max\sum_{i=1}^{D}l\left|x\left(t_{n}\right),f\left(\alpha \left(t_{n}\right)\right)\right|. \end{array}$$

In the Internet of Things (IoT), time series prediction can be used to help make intelligent decisions in areas as diverse as industry, agriculture transport, and mobility, thanks to the huge amount of time-series data collected. The implementation of time series prediction requires the support of time series complementary algorithms. The two complement each other and are indispensable. The virtual simulation system uses relevant hardware equipment to carry out various activities in the constructed virtual world with the help of virtual simulation technology so that students can carry out practical training in a relatively real environment and complete the practical training with the help of the virtual simulation system. Teaching has a better effect.

**Figure 2:**
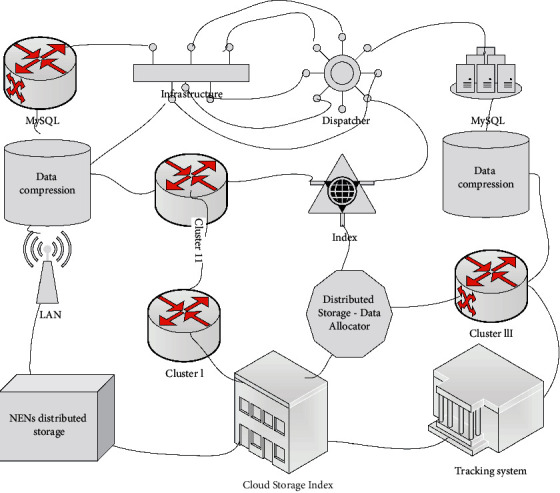
MEN scenario storage collaboration architecture diagram.

With the development of information technology and the integration of information technology and subjects, the presentation of teaching content is bound to change, and so will the way students learn, the way teachers teach, and the way teachers and students interact with each other. Only in this way will students' learning and development be able to adapt to the ever-changing information technology environment and truly realize the all-around development of human beings. In the traditional teaching environment, teachers are the main disseminators of knowledge, and as the old saying goes, “there is a sequence of knowledge, and there is a specialization in the field.” This means that the single model of learning and teaching is no longer suitable for the development of education and teaching, and those diverse ways of learning and teaching need to be effectively integrated into daily teaching activities, as shown in Figure 2.

While the above frameworks are functionally complete, considering various scenarios such as fault recovery and data types, the storage cooptimization in the MEN scenario adapts the commonly used distributed storage frameworks to data storage in the IoT environment. IoT systems are more likely to have upstream endpoint data storage, and this type of data is characterized by small individual data lengths, relatively large data volumes, and high query frequencies, so the MEN scenario adds a few optimizations to the conventional distributed storage framework by first compressing the data into binary data streams to increase the data storage capacity of a single node before storing it and often using cloud-based indexing to record the data. The node number where the data is stored and where it is located in this node is used to improve the speed of querying the data by optimizing index queries. Virtual: build a virtual situation with the help of advanced science and technology; simulation: build a realistic three-dimensional effect in three-dimensional space and complete some complex skills and operation points through experience, demonstration, and operation.

There are various reasons for the low level of information technology application. On the one hand, as a long-standing employment-oriented secondary school, many teachers in this school have not fully adapted to the change from employment to taking the GCE, and culture classes have not yet received sufficient attention. On the other hand, English teachers capable of undertaking GCE education have not yet grown up, and there is a clear shortage of teachers in terms of both quantity and quality. To solve the shortage of English teachers, the school has hired some young English teachers from outside, but some of these teachers are recent graduates with no work experience at all, and some are only teaching as part-time jobs, with varying teaching standards, and the external teachers do not participate in teaching and research activities and cannot take part in school teaching competitions, which is very detrimental to improving the standard of English teaching in our school.

One of the features of the edge control system is that it sinks the computing tasks originally placed in the cloud server to the edge layer closer to the user side, which makes the edge control system unique in handling low latency services [17]. However, the massive amount of data and services in the IoT can often put a high load on edge node processing. In MEN scenarios, once too many tasks are piled up on the node, it is difficult to respond quickly to the services coming from behind, which is undoubtedly a huge impact for time-sensitive services. The single edge node functional framework shows that task scheduling is more important than data reception, computation, and storage in the processing of data. That is, the node first selects the more time-sensitive tasks to be processed before processing them.

### 3.2. Construction of a Multiple Intelligence Model for Teaching English Informatics

A better teaching effect can only be achieved when the students are active. The creation of students' autonomous learning environment is the key to the cultivation of students' autonomous learning. The subjective initiative generated by students is related to students' study habits. It is human nature to love to play, so the virtual simulation technology is gamified, and according to the relevant principles of gamified teaching, the gamified teaching model and strategy implementation are designed so that students can complete their learning independently without supervision. In the process of teaching English informally, and particularly in the process of computer-assisted English teaching, it is a form of individual teaching in which students can take the initiative to control the content and pace of their learning, and each student can make the most appropriate choice according to his or her actual situation, which is fully in line with the principle of teaching according to ability. Any form of teaching is a bilateral activity consisting of the teacher's teaching and the student's learning. Computer-assisted English teaching is precisely what brings teachers and students together effectively, forming an interactive transmission process by its medium of transmission, allowing students to give full play to their main role. This active thinking and repeated “input-output” process is undoubtedly conducive to the students' main role and thus to the digestion and consolidation of knowledge.

(4) $$\begin{array}{c} T_{\text{avg}}=\sum_{i=1}^{m}T_{\text{pi}}^{3},\\ C_{\text{pi}}=\left\{\begin{array}{cc} 1, & T_{\text{pi}}<T_{\text{RD}},\\ 2, & T_{\text{pi}}\geq T_{\text{RD}}. \end{array}\right. \end{array}$$

**Figure 3:**
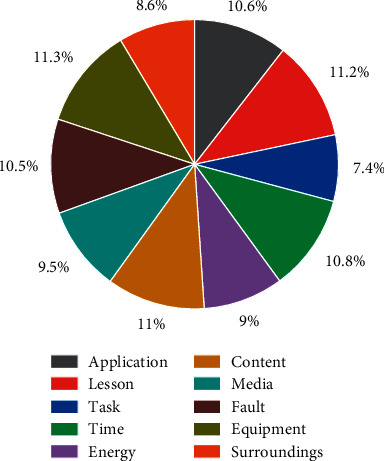
Main difficulties encountered when using modern teaching media.

In the proposed boosted regression model, the residuals in layer *m* can be seen as the amount of variation in the time-series data caused by the *m*th type of attribute factor. Thus, the external component is a combination of different kinds of variation quantities, and the final observed data is in turn the result of the joint action of the external and internal components [18]. Compared with existing methods, the proposed integrated learning-based temporal data modeling and prediction algorithm has the following advantages. It has strong explanatory power and can intuitively reflect the impact of external attributes on the time-series data. The preclassification of attribute features can largely alleviate the dimensional catastrophe problem faced by ANNs and somewhat improve the generalization ability of the prediction model under small samples by multiple simple learners to achieve an approximate learning capability of the DL algorithm, as shown in Figure 3.

85.74% of teachers think that the biggest difficulty they encounter in using IT for teaching is that they do not have good teaching ideas; secondly, if any equipment or teaching software breaks down during teaching, most teachers do not know how to debug it; 61.90% of teachers think that the task of lesson preparation is heavy and they do not have enough time and energy, so it is difficult to use IT to prepare lessons carefully; less than 15% of teachers think that the teaching content is not suitable for using modern information technology [19]. This shows that there is great potential for the application of IT in teaching, but at present, there is still some difficulty in how to apply IT well in teaching and achieve integration of IT with the curriculum. In terms of interest, the novelty of online learning space and the diversification of pictures and videos can provide students with a teaching form that is different from traditional classroom teaching.

As far as classroom order is concerned, an inquiry-based classroom in small groups tests the teacher's ability to control the classroom and whether the students have done an advanced study of the knowledge points before class. In terms of after-class supervision, once online resources and mobile terminals are open to students, the temptations on the Internet are still extremely great for junior high school students in their own time. In terms of the completion of precourse and postcourse revision assignments, as the exercises assigned online are submitted via the Internet and not through paper reports, students have various reasons to excuse themselves from submitting them or even some do not complete them for various reasons, resulting in low completion rates and unsatisfactory completion of assignments [20]. Students who are used to answering on paper do not take the oral practice in the online learning space seriously and ignore online assignments because they feel that the final assessment is still a paper. Parents supervise after-school assignments, and there are various problems once parents do not supervise them properly, online assignments are more conducive to students using online resources to search for answers, and the authenticity of the completed assignments is open to scrutiny. Although the descriptions of the online learning space are different, the researchers all emphasize that the learning space supported by information technology provides a special online learning environment for teachers and students and provides resources sharing, course learning, online testing, class hours for teachers and students' feedback, and so on.

(5) $$\begin{array}{c} S_{\text{th}}=\alpha \cdot \text{Mem}\left[m\right]\left[C_{\text{idel}}\right],\\ S_{\text{th}}=\alpha^{2}C_{\text{idel}}. \end{array}$$

Each piece of knowledge content has a complete system, how to use the virtual simulation for preclass flip, which is similar to the efficient use of virtual simulation teaching, preclass first learning to teach so that students follow the task of independent learning broadens the three-dimensional space and time of the classroom, both time-saving and efficient, according to the creation of three types of the teaching process that encompasses the entire IoT skills teaching. The three types of processes are described in detail in the following section, which ultimately makes the virtual simulation technology better applied.

**Figure 4:**
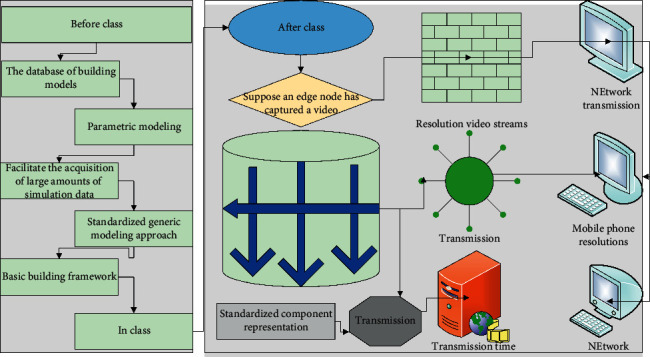
Virtual simulation of three-dimensional teaching.

At the same time in the teaching evaluation, more kinds, characteristics are distinct, students prefer different, distinct personalities, the use of scientific evaluation methods improves the validity and reliability of the evaluation, in the evaluation method using long-term, personalized line point combination of the way to develop classroom participation in the quality of the combination of homework, the current main process evaluation and overall effect evaluation, which process assessment is the most difficult to operate, can be carried out through information tools information collection, which is also a highlight of the self-developed virtual simulation teaching; that is, the integration of the teaching behavior analysis system used for real-time classroom observation, as shown in Figure 4, reflected in data, which is currently virtual simulation teaching use, is unprecedented.

Suppose an edge node has captured a video through its camera and now needs to transmit this video to a specific user for playback [21]. The transmission time required for direct transmission is long and there are many users to be transmitted, each with different mobile phone resolutions. So, the video is now processed on this edge node into different resolution video streams, which are more suitable for network transmission. It is necessary to take the smart learning environment as the technical support, the smart learning as the fundamental cornerstone, and the smart teaching method as the catalysis and promotion.

The file records the transmission rate information between two edge nodes. Assuming that EN5 is the CEN at this point and the number of edge nodes deployed in the environment is 10, then the data in the red box is selected as the environment configuration part of the algorithm input. The basic information of the environment is then configured. To ensure the accuracy of the algorithm, the number of subtasks recorded in the environment is relatively large (over 100) and the number of nodes deployed is relatively large (15); to examine the performance of the algorithm under different circumstances, for example, different numbers of subtasks can be selected to construct different original tasks, different numbers of edge nodes can be deployed, or different edge nodes can be selected as CENs, with each permutation forming a different environment.

Only by interacting with each other can you have endless fun; you chase after me and cultivate the atmosphere of learning. Cooperative learning is currently used in the study of the law. The teaching design of the course is inseparable from group cooperation, so it should also be reflected in the virtual simulation technology. The trajectory and results left by students operating the virtual simulation are recorded. Similar games will have a score record and give necessary feedback, so the interaction between students and students can be reflected in the position between the individual student, the study group, and the whole study so that students can achieve a happy atmosphere of chasing each other, and the traces left after the virtual simulation operation can be transmitted online to other students doing the same.

## 4. Analysis of Results

### 4.1. IoT Scenario Model Performance Results

Li Jing (2007) believes that the rapid development of information technology based on computer technology, network technology, and modern communication technology has greatly changed the traditional teaching mode. In an edge control system for IoT applications, the edge nodes will face a more complex environment, so the original security management mechanisms for cloud servers are no longer applicable to the edge nodes. What is more, the edge computing architecture adopted by the edge control system is closer to the user layer, and the communication process between edge nodes involves a lot of data sharing and computational collaboration between services, which are closely related to the users, so the security management of edge nodes in the edge control system becomes an urgent challenge in the development of edge computing.

**Figure 5:**
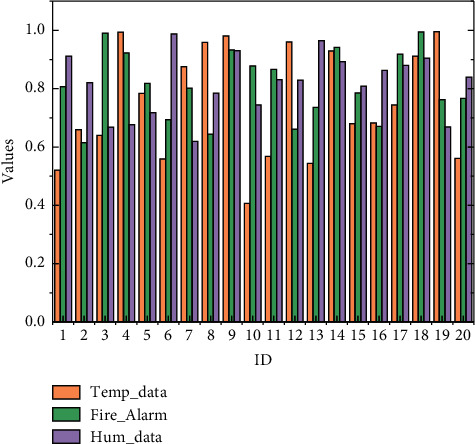
Some of the business data stored in the database MySQL.

This section designs the edge node management platform based on the security management aspects of the edge control system for the edge nodes. The node management platform involves two main aspects: performance monitoring of the edge nodes and node operation and maintenance management of the edge control system. In the algorithm steps involved earlier, it is often necessary to combine the performance usage of the edge nodes themselves for the sake of the business itself. If an edge node is overloaded, it is difficult to complete other tasks assigned to it by the system, so performance monitoring of a single node is necessary, as shown in Figure 5.

In the smart teaching IoT environment, the behavior and data of students in the smart campus environment will be stored in the IoT cloud. Teachers, course managers, or research scholars can directly or indirectly retrieve data from the cloud to formulate wisdom for students' sexual personalized teaching activities. Therefore, it will have a better prediction performance when the variation of electricity load changes is relatively smooth. However, even during test period, the MRE of the Holt–Winter method is still higher than the proposed algorithm. The main reason for this is that the Holt–Winter method cannot fully exploit the external influences. Although the MRE performance of the Holt–Winter method in the test period is the best among the reference algorithms since a real-lifetime series cannot be statistically smooth and it always has abrupt changes caused by external factors, the MARE performance of the Holt–Winter method is poorer, up to 12.77%, compared to 4.42% for the proposed method, which is also consistent with the previous ADF test results—consistent with the previous ADF test results. It can also be observed that AI techniques, including SVR and MLP, have higher MRE results in both test periods, which further illustrates the poor learning ability of AI-based time series forecasting algorithms on small sample datasets.

**Figure 6:**
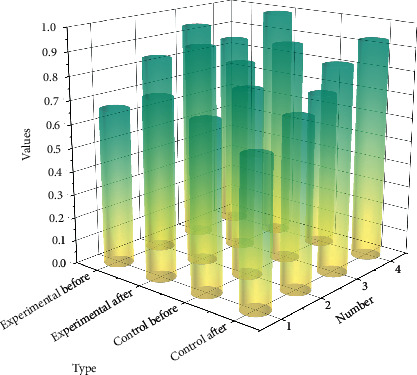
Comparison of knowledge results before and after the experiment.

In addition, the experimental results also show that classical time series analysis methods are not very effective in modeling nonstationary time series and that the MLP algorithm cannot obtain satisfactory results on small sample data sets. In the next sections of this thesis, the corresponding time-series data modeling and analysis methods are investigated for large sample data sets. The algorithm can batch fill in missing values for large-scale time-series data and can solve the problem of filling in the continuous missing cases to ensure the integrity of the data, as shown in Figure 6.

The analysis of the IoT expertise performance was mainly combined with the knowledge system under the senior examination syllabus, comparing the results of the survey on students' knowledge of IoT courses in the two classes after receiving different practical training teaching methods, as shown in the graph. From the data in the table, it was found that the mean value of the experimental group before the experiment was 43.0386 and the standard deviation was 16.78686, while for the students in the control group, the mean value and standard deviation before the experiment were 41.8637 and 11.71201, respectively, *p* = 0.783, indicating that there was no significant difference between the two groups in IoT expertise, no matter who was set as the experimental group or the control group.

Most English teachers adopt a “one-to-many” teaching mode. If English teachers want to control classroom discipline and emphasize personal teaching authority, they can only choose a relatively closed teaching form. The difference in students' knowledge scores before and after the experiment was significant, whereas the mean and standard deviation of the control group before the experiment was 41.8637 and 11.71201 and after the experiment was 50.8637 and 19.67667, respectively, *p* = 0.029, indicating that the difference in students' knowledge scores before and after the experiment in the control group was significant.

### 4.2. Experimental Results of the Multiple Intelligence Model of English Informatics Teaching

Following the mechanisms of pupil's development and of the teacher's role in pupil's development, a new understanding of the structure of teaching and learning activities should be developed. Although teaching made up of both teaching and learning activities, teaching and learning are not two parallel or equivalent elements that make up teaching and learning activities; there are important functional differences and special links between the two. In the teaching process, which is based on the development of student's physical and mental qualities, students' active and effective learning is the ontological (purpose) activity of the teaching process, while the teacher's teaching is the conditional (means) activity that causes students' active participation in learning activities and facilitates their effective completion of the learning process. It is easy to value the status and role of the teacher in the teaching and learning process and to underestimate the status and role of the student in the teaching and learning process. Therefore, the concept of “learning as the center and teaching as a service to learning” has strong relevance and practical guidance value in China.

**Figure 7:**
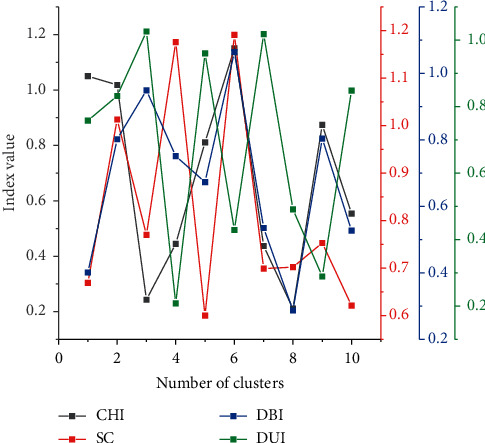
Curves of the four clustering indicators with the number of clusters.

But this kind of teaching stays more at the level of resource utilization and the use of information technology is not sufficient. Classroom teaching Case 2 shows us that the integration of information technology and teaching is more successful. Information technology plays a full role not only in the use of resources but also in the organization, management, and evaluation of teaching. The aim of learning is no longer merely to master knowledge, but more to solve problems. To a certain extent, real quality education has been achieved. Of course, this kind of education and teaching reform needs more institutional support and more human and material resources, as shown in Figure 7.

The clustering results show that the shape of the flow profile of the road section is generally consistent with the shape of the flow profile of other road sections within the same group, demonstrating DeepCluster's ability to extract shape-based features.

**Figure 8:**
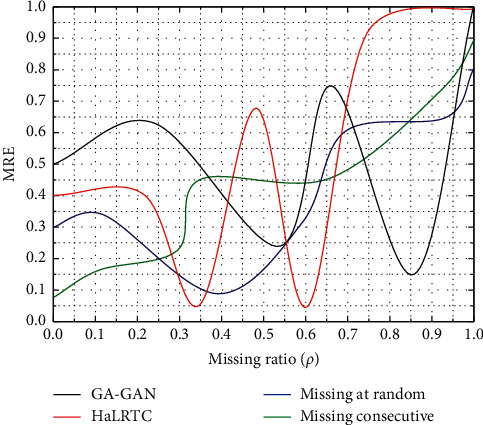
Experimental results.

This is because indications of future short-term changes can often be obtained by looking at changes in recent data. That is, the closer the inputs used for prediction are to the prediction point, the more information the model obtains about the prediction point. Thus, in the case of relatively slow and steady changes in flow rates, the PM is a good predictor of the trend in traffic flow rates for all forecast ranges. However, when the flow rate changes rapidly, the input to predict the next 10-minute or 15-minute flow rate has less information about this sudden change than the input to predict the next 5-minute flow rate. Therefore, with large prediction intervals, it is difficult for the model to capture information and indications of sudden changes in velocity at the outset. However, after gradually acquiring information on the latest change in velocity, the model can be adjusted in a short time to keep up with the change. It can also be seen from the graph that the model's prediction error for the recovery period before 7:30 is significantly smaller than that for the period after the onset of congestion, as shown in Figure 8.

The primary and secondary school network cloud platform has provided strong support for this large-scale online education. It is expected that the platform will be further improved after this epidemic to bring into play the intensive effect and provide a realistic lead for the development of education informatization. Through the interface with the national primary and secondary school students' school registration information management system, individual, class, and school and regional students' online learning performance assessment reports are formed to realize individual comprehensive quality assessment and regional and school academic quality assessment, providing support for examination admissions and teaching improvement, further playing the “revolutionary” role of information technology. English teachers themselves have accumulated certain teaching experience in front-line teaching and have worked out teaching rules that are in line with the school and student situations. Teachers continue to improve their concept of information-based teaching, strengthen their sense of professional identity and belonging, and constantly update their knowledge of English information-based teaching from the cognitive aspect.

## 5. Conclusion

In this paper, the virtual scene of the Internet of Things is applied to English informatization teaching, which breaks the traditional indoctrination teaching mode and changes from teaching-centered teaching to learning-centered teaching. Use the skills of experience, demonstration, interaction, and assessment in the virtual environment of the Internet of Things to stimulate students' interest in active learning and increase the interest of English classroom teaching. This paper proposes a centralized multitask scheduling method for the problem of how to reduce the processing delay of IoT scenarios. This method first designs a multilevel scheduling framework that combines process scheduling and thread scheduling. Process scheduling adopts a preemptive static priority scheduling algorithm. Regarding thread scheduling for the same kind of business with high concurrency, this paper proposes a task urgency factor and designs a thread scheduling algorithm based on dynamic priority. The experimental results show that the multilevel scheduling method plays a significant role in ensuring the timeliness of tasks and can reduce the delay problem to a certain extent. The approach starts by building a framework for distributed collaborative computing using an asynchronous message queue MQ, which allows multiple nodes to collaborate on a single task through task splitting. In the task offloading module of the framework, a task offloading decision algorithm based on a value-constrained multi 0–1 backpacking model is designed to minimize the task processing latency with an optimal offloading solution. System test results show that the proposed distributed computing framework and offloading decision algorithm can significantly reduce the processing latency of large tasks.

## Data Availability

The data used to support the findings of this study are available from the corresponding author upon request.
